# Transcriptome Analysis of Maize Immature Embryos Reveals the Roles of Cysteine in Improving *Agrobacterium* Infection Efficiency

**DOI:** 10.3389/fpls.2017.01778

**Published:** 2017-10-17

**Authors:** Yan Liu, Zhiqiang Zhang, Junjie Fu, Guoying Wang, Jianhua Wang, Yunjun Liu

**Affiliations:** ^1^College of Agronomy and Biotechnology, China Agricultural University, Beijing, China; ^2^Institute of Crop Sciences, Chinese Academy of Agricultural Sciences, Beijing, China

**Keywords:** *Agrobacterium*, infection efficiency, cysteine, maize embryo, transcriptome

## Abstract

Maize *Agrobacterium*-mediated transformation efficiency has been greatly improved in recent years. Antioxidants, such as, cysteine, can significantly improve maize transformation frequency through improving the *Agrobacterium* infection efficiency. However, the mechanism underlying the transformation improvement after cysteine exposure has not been elucidated. In this study, we showed that the addition of cysteine to the co-cultivation medium significantly increased the *Agrobacterium* infection efficiency of hybrid HiII and inbred line Z31 maize embryos. Reactive oxygen species contents were higher in embryos treated with cysteine than that without cysteine. We further investigated the mechanism behind cysteine-related infection efficiency increase using transcriptome analysis. The results showed that the cysteine treatment up-regulated 939 genes and down-regulated 549 genes in both Z31 and HiII. Additionally, more differentially expressed genes were found in HiII embryos than those in Z31 embryos, suggesting that HiII was more sensitive to the cysteine treatment than Z31. GO analysis showed that the up-regulated genes were mainly involved in the oxidation reduction process. The up-regulation of these genes could help maize embryos to cope with the oxidative stress stimulated by *Agrobacterium* infection. The down-regulated genes were mainly involved in the cell wall and membrane metabolism, such as, aquaporin and expansin genes. Decreased expression of these cell wall integrity genes could loosen the cell wall, thereby improving the entry of *Agrobacterium* into plant cells. This study offers insight into the role of cysteine in improving *Agrobacterium*-mediated transformation of maize immature embryos.

## Introduction

An efficient crop transformation method is one of the prerequisites for creating transgenic crops. At present, widely used transformation methods include the particle bombardment method and the *Agrobacterium*-mediated method. Compared with particle bombardment, *Agrobacterium*-mediated transformation has the advantages of simple operation, low cost, low foreign gene copy number, and the stable inheritance of foreign genes. Through whole genome mutation and phenotype screening, many *Agrobacterium* genes were identified that play key roles in the transformation process. One collection of genes involved in excising DNA and integrating it into a foreign genome is the *Vir* region. It is well known that phenols or jasmonic acid can induce the expression of *Agrobacterium Vir* genes under the acidic environments. The transcriptome and proteome analysis of *Agrobacterium* treated with acetosyringone, illustrated that Vir proteins have different expression response mechanisms (Cho and Winans, 2005; Lai et al., 2006). It is known that *VirA* and *VirG* maintain constant expression levels in *Agrobacterium*. When stimulated with an outside signal, the protein kinase VirA can phosphorylate VirG and increase its expression; As a transcription factor, VirG proteins activate the expression of other *Vir* genes (Brencic and Winans, 2005). The T-complexes formed between VirD2 and T-DNA enter the plant cell, where the T-complexes can now bind the other Vir proteins (VirE2, VirE3, VirD5, and VirF) to form a super complex. This super complex can enter the plant cell nucleus and release the T-DNA for integration into the plant genome (Gelvin, 2010; Pitzschke and Hirt, 2010).

Plant genes also participate in the process of *Agrobacterium* mediated transformation (Pitzschke and Hirt, 2010). Through screening mutants resistant to *Agrobacterium tumefaciens* transformation, more than 129 genes have been identified to influence the Arabidopsis transformation by *A. tumefaciens* (Zhu et al., 2003b). In tobacco cells, the *Agrobacterium* VirE2 proteins have been shown to interact with tobacco VIP1 and VIP2 proteins, and VIP1 overexpression significantly improved the transformation efficiency (Tzfira et al., 2002). It was shown that VIP1 is a substrate of the MPKK signaling pathway in the plant, where signals are transferred from the cytoplasm to the nucleus after phosphorylation by MPKKK, mediating the entry of the Vir protein and T-DNA T-complex (Djamei et al., 2007). The plant genes *AtAGP17*, encoding α galactosan (Gaspar et al., 2004) and *CslA-09* gene, encoding cellulose synthase (Zhu et al., 2003a), play roles in *Agrobacterium* attachment to plant cells. The plant transporter β3 has been shown to play an important role in the entry of Vir and T-DNA into the nucleus (Zhu et al., 2003b). On the contrary, the Arabidopsis MTF1 protein has been shown to negatively affect the attachment of several *Agrobacterium* strains to roots, thereby decreasing the transformation efficiency (Sardesai et al., 2014). Other plant proteins, such as, SUPPRESSOR OF G2 ALLELE OF SKP1 (SGT1) and heat shock protein 90.1, are required for *Agrobacterium*-mediated transformation (Anand et al., 2012; Park et al., 2014). Many plant genes play important roles in the controlling of callus formation and organ re-differentiation, including AUXIN RESPONSE FACTORs, LEAFY COTYLEDON1, WUSCHEL, BABY BOOM, AGAMOUS-LIKE15, and SOMATIC EMBRYOGENESIS RECEPTOR KINASE (Altpeter et al., 2016). Overexpression of maize *Baby boom* (*Bbm*) and maize *Wuschel2* (*Wus2*) genes have been shown to achieve high transformation efficiency in numerous recalcitrant maize inbred lines (Lowe et al., 2016; Mookkan et al., 2017).

To achieve high efficient *Agrobacterium*-mediated maize transformation, the plant genotypes, explants, *Agrobacterium* strains, co-cultivation medium and other factors should be taken into consideration. The milepost type of work on *Agrobacterium*-mediated maize transformation comes from Ishida et al. (1996), where a super binary vector carrying the *virB, virC*, and *virG* genes, was used to infect the immature embryos of maize inbred line A188, achieving 5–30% transformation efficiency. Maize transformation efficiency has also been improved by treating immature embryos with heat prior to *Agrobacterium* infection (Hiei et al., 2006). In aforementioned maize transformation systems, *in vitro* tissue culture was maintained in dark conditions. Cho et al. (2014) established a high frequency maize transformation protocol for recalcitrant maize inbred lines, by initiating and maintaining green tissues under dim light condition.

Antioxidants such as, cysteine, dithiothreitol (DTT), glutathione, and ascorbic acid can reduce tissue necrosis in explants during plant transformation. Cysteine is often used to improve the transformation efficiency of soybean (Olhoft and Somers, 2001; Zeng et al., 2004). In sugarcane transformation, usage of the media containing ascorbic acid and cysteine has been shown to offer higher transformation efficiency (Enríquez-Obregón et al., 1998). It has also been shown that the anti-oxidative compounds lipoic acid enhances the *Agrobacterium*-mediated transformation of MicroTom and soybean by mitigating oxidative stress (Dan et al., 2015). The addition of cysteine or DTT in co-cultivation medium has also been shown to significantly improve maize transformation efficiency (Frame et al., 2002; Vega et al., 2008).

Although it has been shown that cysteine can improve the transformation efficiency, the mechanism has not been elucidated. In this study, we showed that cysteine could improve *Agrobacterium* infection efficiency in the maize inbred line Z31 and hybrid line HiII. We further investigated the mechanism behind cysteine-related infection efficiency increase using transcriptome analysis. The results showed that the up-regulated genes were mainly involved in the oxidation reduction process, and the down-regulated genes were mainly involved in the cell wall and membrane metabolism. We hypothesize that cysteine loosens the cell wall by modifying the expression of genes involved in cell wall metabolism and membrane metabolism, i.e., aquaporin and expansin genes, thereby improving the entry of *Agrobacterium* into plant cells.

## Materials and methods

### Plant material and *Agrobacterium* strain

Inbred line Z31 and hybrid line HiII were used in this study. Maize plants were grown in the greenhouse under a 16/8h light/dark cycle at 20–25°C. The *Agrobacterium* strain used in this study was EHA105 containing the binary vector pCambia3301 that carries a T-DNA with a beta-glucuronidase (*gus*) gene.

### Immature embryo treatment

The immature embryos were collected from the maize ears 10–12 days after pollination. The ears were immersed in filter-sterilized MS-INF (4.1 g L^−1^ MS medium, 0.115 g L^−1^ L-Proline, 36 g L^−1^ glucose, 68.5 g L^−1^ sucrose, 100 μM acetosyringone, pH 5.4). The *Agrobacterium* strain was cultured in autoclaved liquid YEP medium (10 g L^−1^ tryptone, 10 g L^−1^ yeast extract, 5 g L^−1^ NaCl, 50 mg L^−1^ rifampicin, 50 mg L^−1^ kanamycin, pH 7.0) overnight shaking at 220 rpm at 28°C. The *Agrobacterium* was collected by centrifuge and re-suspended to OD_600_ = 0.4 in the MS-INF medium. The collected embryos were heated in a 45°C water bath for 2 min, and then infected with the *Agrobacterium* suspension for 5 min. After infection, the immature embryos were transferred onto MS CO-medium (4.1 g L^−1^ MS medium, 0.7 g L^−1^ L-Proline, 10 g L^−1^ glucose, 20 g L^−1^ sucrose, 0.85 mg L^−1^ silver nitrate, 100 μM acetosyringone, 1.5 mg mL^−1^ 2,4-D, 3.5 g L^−1^ phytagel, pH 5.8) with or without 100 mg L^−1^ cysteine that was freshly prepared and filter-sterilized. After culturing for 3 days at 25°C under dark conditions, the immature embryos were collected, and stored at −70°C for RNA extraction. Ultimately, four treatments were applied to the maize embryos: (1) no heat and no cysteine-infused media, (2) no heat and cysteine-infused media, (3) heat and no cysteine-infused media, and (4) heat and cysteine-infused media.

### Analysis of transient GUS expression

Three days after growth on media with or without cysteine, the transient *gus* expression was analyzed using a histochemical GUS assay. The embryos were immersed in the GUS staining solution (Jefferson et al., 1987) and incubated overnight at 37°C. The level of transient *gus* expression was assessed based on the number of visible blue foci on the scutellum side of each embryo (Frame et al., 2002).

### Reactive oxygen species determination

The Z31 immature embryos were cultured for 3 days at 25°C in dark condition on media with or without cysteine after infection by *A. tumefaciens* EHA105. The immature embryos (0.1 g) were homogenized with phosphate buffer saline (PBS) solution (pH 7.4), and then centrifuged at 8,000 rpm and 4°C for 30 min, and the supernatant was used for subsequent reactions. The hydrogen peroxide (H_2_O_2_) content was measured using an enzyme-linked immunosorbent assay (ELISA) kit (Tsz Biosciences, Woburn, USA). The superoxide anion content was measured using an ELISA kit (SU-B91178, Kenuodi Biotech, Fujian, China). *In situ* superoxide anion (${\text{O}}_{2}^{-}$) was estimated using the NBT staining method and H_2_O_2_ using the DAB staining method (Dutilleul et al., 2003).

### Transcriptome analysis

Total RNA was extracted from embryos after 3-day co-cultivation with or without cysteine using the RNeasy Plant Mini Kit (Qiagen, Germany). The extracted RNA was run in a 1% agarose gel to assess the integrity of the RNA. The RNA yield and purity were checked using the Nano-drop ND-1000. Poly(A) mRNAs were isolated from the total RNA using oligo (dT) magnetic beads (Illumina, San Diego, CA). RNA fragmentation, cDNA synthesis, and PCR amplification were performed according to the Illumina RNA-Seq protocol. The cDNA libraries were sequenced with a read length of 100 bp (paired-end) using the Illumina HiSeq 2000 System at Berry Genomics (Beijing, China). The experiment was performed with four biological replicates.

### Data analysis

The obtained raw data were processed with Perl scripts to remove the adaptor-polluted reads, low-quality reads and reads with the number of N bases accounting for more than 5%. The filtered reads were used for the quality and quantity analyses. The TopHat software v2.0.12 was used to map the clean reads to the B73 RefGen_V3 genome (www.maizegdb.org).

FPKM (fragments per kilobase of transcripts per million fragments mapped) was calculated to estimate the expression level of each sample. The Cuffdiff program within the Cufflinks software was used to identify the differentially expressed genes (DEGs) based on the following thresholds: |fold change| ≥ 2 and FDR cut-off < 0.01.

MapMan software (version 3.5.1R2; http://mapman.gabipd.org/) was used to analyze the metabolic pathways according to the top hits to the maize genome database. In addition, agriGO (http://bioinfo.cau.edu.cn/agriGO/) was used to estimate the biological process, molecular function, and cellular component for each DEGs.

### Quantitative real-time PCR analysis

The first-strand cDNA synthesis was performed with the M-MuLV reverse transcriptase (Promega) using total RNA as the template. For the quantitative real-time PCR (qRT-PCR), 1 μL of cDNA was mixed with 2 × SYBR premix ExTaq (Takara), 0.2 μM forward primer, 0.2 μM reverse primer, and 0.4 μL 50 × ROX in 20 μL of reaction mixture. The qRT-PCR was conducted using the ABI 7300 system with the following protocol: 95°C for 2 min, 40 cycles at 95°C for 5 s, 58°C for 30 s, and 72°C for 31 s. The relative transcriptional levels were calculated using the 2^−ΔΔCt^ method (Livak and Schmittgen, 2001) with *actin* as a housekeeping gene.

## Results

### Heat and cysteine treatments improve the *Agrobacterium* infection efficiency of maize embryos

**Figure 1:**
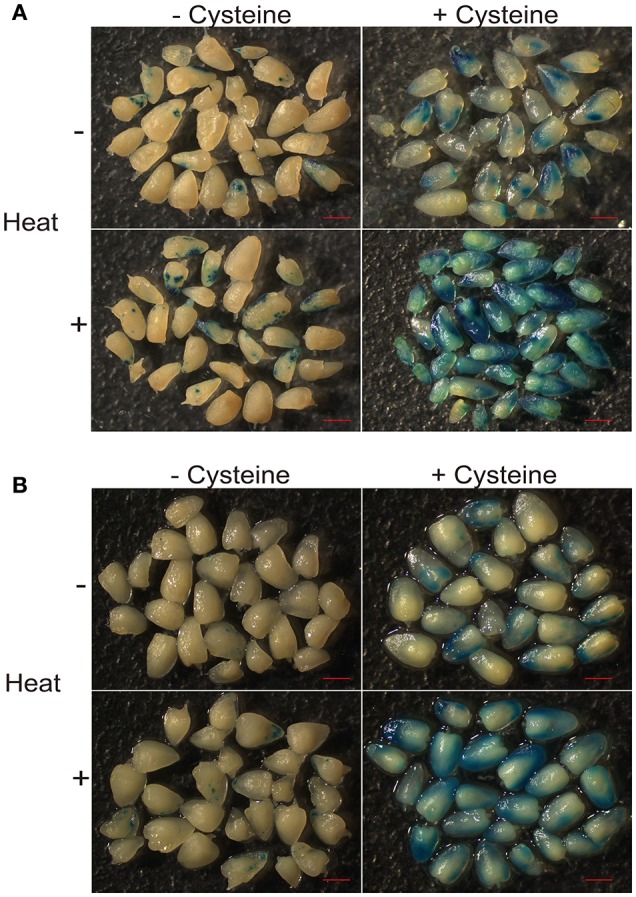
Heat treatment and cysteine effect on *Agrobacterium* infection efficiency. **(A)** Maize hybrid HiII; **(B)** Maize inbred line Z31.

**Figure 2:**
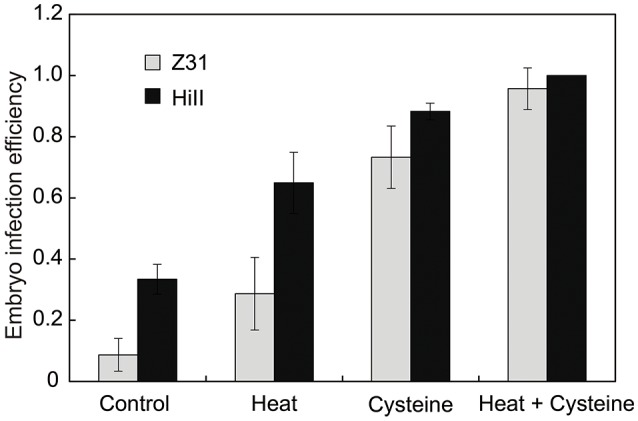
The embryo infection efficiency. Data was shown as the average ± s.e. of three independent experiments.

It has been reported that the addition of cysteine in medium can significantly improve transformation efficiency in the maize hybrid HiII (Frame et al., 2002; Vega et al., 2008). Heat shock is also an efficient way to improve the transformation efficiency (Hiei et al., 2006). To test the transformation efficiency in the maize elite inbred line Z31 and maize hybrid HiII, we combined both strategies, including an embryo heat treatment prior to *Agrobacterium* infection and the addition of cysteine in the co-cultivation medium. The *Agrobacterium* strain EHA105 carrying the binary vector pCambia3301 was used for maize embryo transformation with the *gus* gene. After culturing with or without cysteine for 3 days, the transient GUS expression was analyzed by histochemical GUS assays. For both Z31 and HiII materials, heat treatment somewhat increased infection efficiency. Compared with heat treatment, the addition of cysteine in the co-cultivation medium had greater effects on the infection efficiency (Figures 1, 2). High infection efficiency was observed for the HiII material, compared to maize inbred line Z31 (Figures 1, 2).

### Reactive oxygen species accumulation in *Agrobacterium*-infected embryos

**Figure 3:**
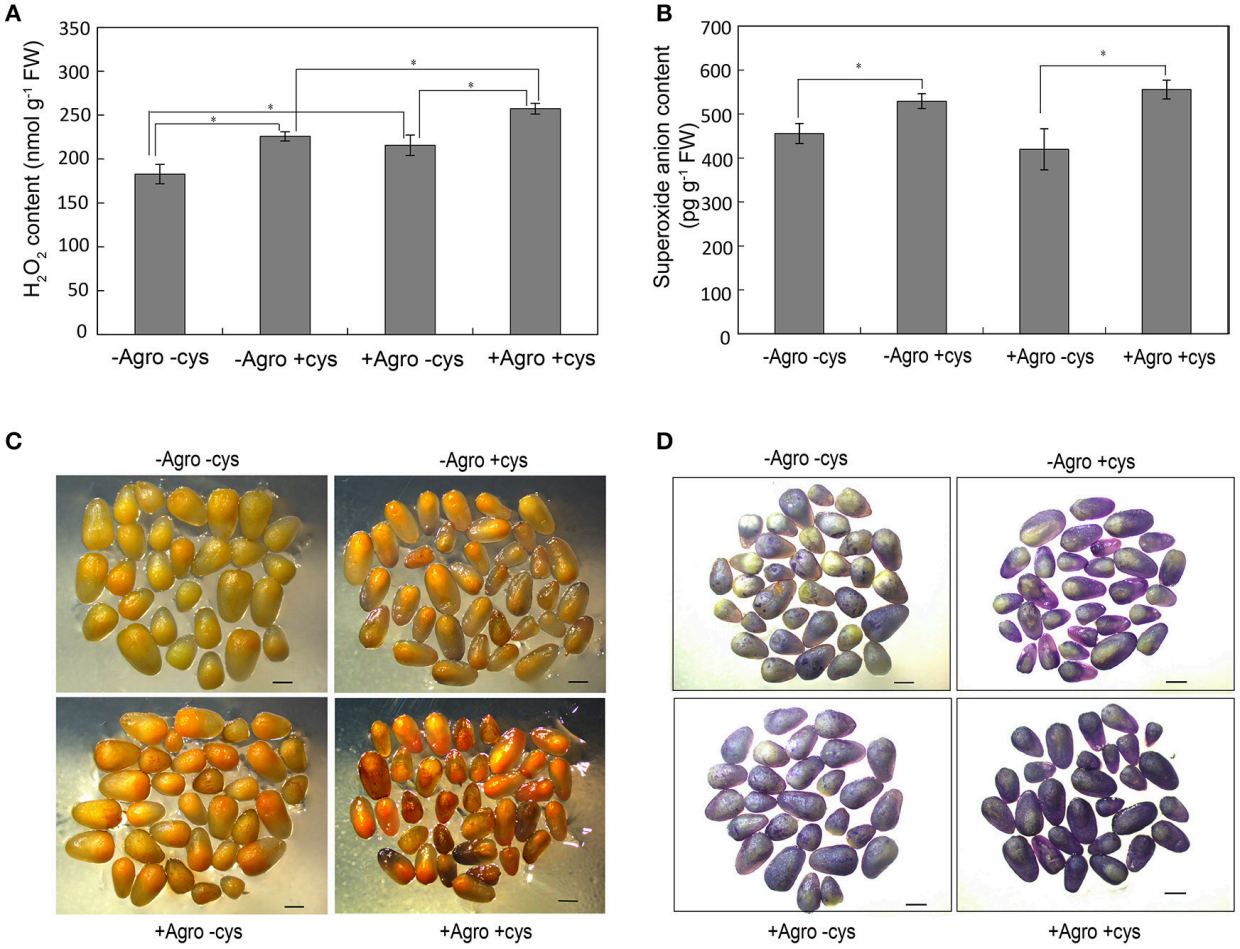
Reactive oxygen species contents in maize embryos. The Z31 immature embryos were infected or not infected by *Agrobacterium tumefaciens* EHA105, then cultured for 3 days at 25°C in dark condition on co-cultivation media with or without cysteine. H_2_O_2_ **(A)** and ${\text{O}}_{2}^{-}$ **(B)** contents in embryos were measured and the data was shown as average ± s.e. of three independent experiments. Asterisks indicated the significant difference at *P* < 0.05 level. *In situ* H_2_O_2_ **(C)** and ${\text{O}}_{2}^{-}$ **(D)** was estimated using the DAB and NBT staining method, respectively.

The reactive oxygen species hydrogen peroxide (H_2_O_2_) and superoxide anion (${\text{O}}_{2}^{-}$) levels were measured in the *Agrobacterium*-infected embryos. *Agrobacterium* infection stimulated the accumulation of H_2_O_2_ in embryos grown on co-cultivation medium with or without cysteine (Figures 3A,C), whereas did not affect the accumulation of ${\text{O}}_{2}^{-}$ (Figures 3B,D). To our surprise, the addition of cysteine in the co-cultivation medium significantly increased the accumulation of H_2_O_2_ and ${\text{O}}_{2}^{-}$ in embryos (Figure 3). These results indicated that cysteine may improve the *Agrobacterium* infection efficiency not just as an antioxidant, but with other mechanism.

### Maize embryo transcriptome profiling

To investigate the mechanism of cysteine-related transformation efficiency improvement, we performed transcriptome analysis of the maize embryos cultured on the medium with or without cysteine. The experiment was performed with four independent biological replicates. We obtained 7,432,161–15,904,122 reads from the HiII embryo samples and more than 78.73% of the reads were mapped to the B73 reference genome. We obtained 20,119,176–24,789,278 reads from the inbred line Z31 embryo samples and 73.66% of the reads were mapped to the B73 reference genome (Table 1). Maize has approximately 30,000 genes and the sequencing depth we acquired was enough for subsequent analysis.

**Table 1:** RNA-seq reads of maize embryo mapped to the maize B73 RefGen_V3 genome.

**Maize lines**	**Sample**	**Repeat**	**Total reads**	**Mapped reads**	**Mapping rate (%)**
HiII	Cys0	1	11,098,852	8,706,654	79.0
	Cys0	2	7,432,161	5,528,821	74.4
	Cys0	3	14,191,549	11,331,100	79.8
	Cys0	4	10,107,230	8,049,452	79.6
HiII	Cys100	1	11,326,661	8,739,394	77.2
	Cys100	2	15,881,554	12,726,762	80.1
	Cys100	3	12,195,634	9,807,213	80.4
	Cys100	4	13,405,438	10,636,789	79.3
Z31	Cys0	1	24,789,278	18,229,005	73.5
	Cys0	2	22,532,778	16,268,201	72.2
	Cys0	3	21,844,814	16,133,350	73.9
	Cys0	4	21,579,742	15,987,483	74.1
Z31	Cys100	1	20,119,176	14,818,590	73.7
	Cys100	2	21,478,014	16,045,958	74.7
	Cys100	3	24,494,505	17,793,224	72.6
	Cys100	4	23,553,167	17,615,606	74.6

**Figure 4:**
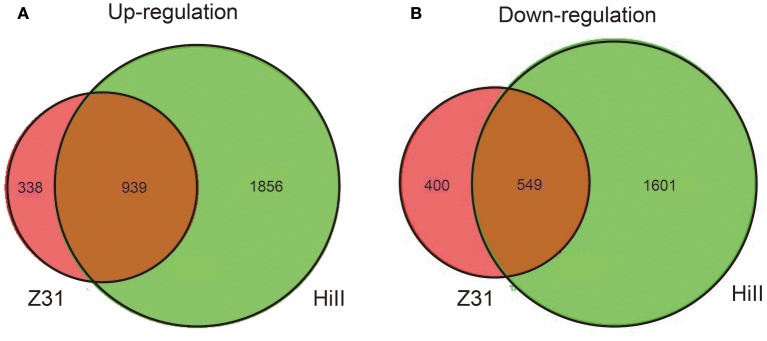
Venn diagram of transcriptome between maize hybrid HiII and inbred line Z31. **(A)** Shared and unique up-regulated DEGs in HiII and Z31 maize lines. **(B)** Shared and unique down-regulated DEGs in HiII and Z31 maize lines.

In this study, |fold change| ≥ 2 and FDR cut-off < 0.01 was set as the threshold to select for DEGs. A total of 2,795 and 1,277 genes were up-regulated in the cysteine treatment in Z31 and HiII, respectively. Among them, 939 genes were up-regulated in both Z31 and HiII. A total of 2,150 and 949 genes were down-regulated in the cysteine treatment in Z31 and HiII, respectively. Five hundred and forty-nine genes among them were down-regulated in both Z31 and HiII (Figure 4). To confirm the accuracy of the transcriptome analysis results, the transcripts of 11 genes were analyzed using qRT-PCR. The primers for these genes are shown in Table S1. The qRT-PCR results showed that the expression profiles of these genes were consistent with the RNA sequencing data (Table 2), indicating that our RNA sequencing results were valid.

**Table 2:** Confirmation of the transcriptome results by qRT-PCR.

**Gene**	**Gene annotation**	**Fold change**	
		**RNA-Seq**	**qRT-PCR**
GRMZM2G036708	Cysteine synthase	6.44	14.22
GRMZM2G170017	Carbonyl reductase 1	13.40	19.97
GRMZM2G087875	Cytochrome P450 CYP81A1	25.03	31.44
GRMZM2G132875	NAD(P)H-dependent oxidoreductase	8.84	4.90
GRMZM2G074743	Alternative oxidase	18.04	6.99
GRMZM2G443445	Mannitol dehydrogenase	6.18	9.23
GRMZM2G097641	Sucrose-phosphatase 2	7.04	8.30
GRMZM2G070322	Systemin receptor SR160	2.66	2.27
GRMZM2G025105	Polygalacturonase inhibitor	5.46	2.60
GRMZM2G166944	Xyloglucan endotransglucosylase/hydrolase protein 23	3.01	1.57
GRMZM2G025190	Glutathione S-transferase GSTU6	14.84	7.84

### Metabolism overview of differentially expressed genes in maize embryos

**Figure 5:**
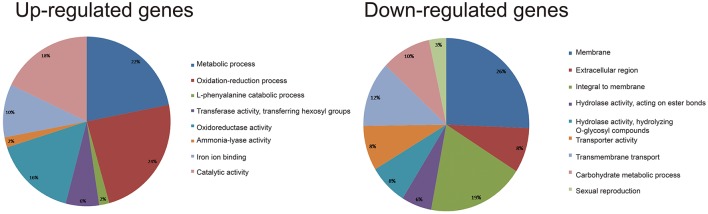
GO analysis of the shared up-regulated **(left)** and down-regulated **(right)** DEGs in HiII and Z31 maize embryos.

To elucidate how cysteine influences *Agrobacterium* infection efficiency, the putative function of DEGs was analyzed using Blast2GO. We performed GO analysis of the up-regulated and down-regulated genes in HiII and Z31. Due to fact that HiII and Z31 had different responses to the cysteine treatment, the common DEGs in both materials were used to elucidate the mechanism. The common up-regulated genes were divided into the following categories: metabolic process, oxidation-reduction process, L-phenylalanine catabolic process, transferase activity, transferring hexosyl groups, oxidoreductase activity, ammonia-lyase activity, iron ion binding, and catalytic activity (Figure 5). Results showed that 24% of the common up-regulated genes belonged to the oxidation-reduction process and 16% of the genes belonged to the oxidoreductase activity process. These two processes have important roles in the regulation of the redox balance of the plant cells. As a reducing agent, cysteine may induce the expression of the redox genes and lead to high metabolic activity in plant cells, protecting them against bacterial invasion.

The common down-regulated genes were divided into the following categories: membrane metabolism; extracellular region; hydrolase activity; acting on ester bonds; hydrolase activity; hydrolyzing O-glycosyl compounds; transporter activity; transmembrane transport; carbohydrate metabolic process; and sexual reproduction process. The down-regulated genes were mainly related to membrane metabolism, with ~45% of the shared DEGs occupying this category (Figure 5). Additionally, 12% of the down-regulated genes were related to the transmembrane transport category. These results indicated that cysteine might improve the infection efficiency by modifying the cell wall membrane.

### Genes involved in infection efficiency improvement

#### Genes involved in the oxidation-reduction process

Among the common up-regulated genes, many are involved in the oxidation-reduction process or oxidoreductase activity process and metabolic process (Table 3). Cysteine synthase (GRMZM2G036708) is responsible for the final step in biosynthesis of cysteine, catalyzing O3-acetyl-L-serine into L-cysteine. Cysteine synthase also participates in the selenoamino acid metabolism and sulfur metabolism. It has been shown that overexpression of cysteine synthase in transgenic tobacco can increase tolerance to sulfur dioxide and sulfur (Noji et al., 2001).

**Table 3:** DEGs which involved in the cell wall metabolism.

**Pathway**	**Gene ID**	**Gene anotation**	**Expression pattern**
	GRMZM2G052571	Glutathione S-transferase	Up-regulated
	GRMZM2G056388	Glutathione S-transferase	Up-regulated
Oxidation reduction process	GRMZM2G036708	Cysteine synthase	Up-regulated
	GRMZM2G159587	Glyoxylate reductase	Up-regulated
	GRMZM2G170017	Carbonyl reductase 1	Up-regulated
	GRMZM2G177077	Glucose-6-phosphate 1-dehydrogenase	Up-regulated
	GRMZM2G141473	Aldehyde oxidase-2	Up-regulated
	GRMZM2G169890	Superoxide dismutase	Up-regulated
	GRMZM2G058522	Superoxide dismutase	Up-regulated
	GRMZM2G471357	Peroxidase 52	Up-regulated
	GRMZM2G440208	6-phosphogluconate dehydrogenase	Up-regulated
	GRMZM2G173195	Glycerol-3-phosphate dehydrogenase	Up-regulated
	GRMZM2G090980	Mannitol dehydrogenase	Up-regulated
	GRMZM2G058244	UDP-glucose 6-dehydrogenase	Up-regulated
	GRMZM2G053720	Proline oxidase	Up-regulated
	GRMZM2G074743	Alternative oxidase	Up-regulated
	GRMZM2G479423	Aldose reductase	Up-regulated
	GRMZM2G443445	Mannitol dehydrogenase	Up-regulated
	GRMZM2G099467	Gibberellin 20 oxidase 2	Up-regulated
	GRMZM2G072529	Acc oxidase	Up-regulated
	GRMZM2G102959	Ferredoxin nitrite reductase	Up-regulated
Membrane integrity and transport	GRMZM2G041980	Aquaporin NIP1-1	Down-regulated
	GRMZM2G392975	Aquaporin PIP1-1	Down-regulated
	GRMZM2G126582	Aquaporin NIP-type	Down-regulated
	GRMZM2G081843	Aquaporin PIP 1-3	Down-regulated
	GRMZM2G041980	Aquaporin NIP1-1	Down-regulated
	GRMZM2G392975	Aquaporin PIP1-1	Down-regulated
	GRMZM2G126582	Aquaporin NIP-type	Down-regulated
	GRMZM2G081843	Aquaporin PIP 1-3	Down-regulated
	GRMZM2G027098	Aquaporin TIP2-2	Down-regulated
	GRMZM2G047368	Aquaporin PIP2-4	Down-regulated
	GRMZM2G178693	Aquaporin PIP2-4	Down-regulated
	GRMZM2G154628	Aquaporin PIP2-4	Down-regulated
	GRMZM2G060922	Aquaporin SIP1-2	Down-regulated
	GRMZM2G159632	Sulfate transporter	Down-regulated
Membrane integrity and transport	GRMZM2G442523	Sugar transport protein 5	Down-regulated
	GRMZM2G063824	Carbohydrate transporter	Down-regulated
	GRMZM2G342907	Sulfate transporter	Down-regulated
	GRMZM2G036448	Amino acid-polyamine transporter	Down-regulated
Cell wall metabolism	GRMZM2G166944	Xyloglucan endotransglucosylase	Up-regulated
	GRMZM2G392125	xyloglucan endotransglucosylase	Up-regulated
	GRMZM2G070271	Probable xyloglucan endotransglucosylase	Up-regulated
	GRMZM2G026980	Xyloglucan endotransglycosylase	Up-regulated
	GRMZM2G070322	Systemin receptor SR160	Up-regulated
	GRMZM2G021621	Expansin-B4	Down-regulated
	GRMZM2G094990	Beta-expansin 1a	Down-regulated
	GRMZM2G414779	Expansin-A31-like	Down-regulated
	GRMZM2G339122	Alpha expansin 1	Down-regulated
	GRMZM2G368886	Alpha expansin 4	Down-regulated
	GRMZM2G148485	Expansin-B15-like	Down-regulated
	GRMZM2G082520	Beta-expansin 1a	Down-regulated
	GRMZM2G013002	Beta expansin8	Down-regulated
	GRMZM2G342246	Beta-expansin 7	Down-regulated
	GRMZM2G021427	Expansin-B3-like	Down-regulated
	GRMZM2G025231	Cellulose synthase 7	Down-regulated
	GRMZM2G178025	Endoglucanase 12-like	Down-regulated
	GRMZM2G453565	Endoglucanase 2-like	Down-regulated
	GRMZM2G147687	Exoglucanase 1	Down-regulated
	GRMZM2G141911	Endoglucanase 4-like	Down-regulated
	GRMZM2G131912	Pectate lyase 8	down-regulated
	GRMZM2G091191	Brassinosteroid-regulated protein BRU1-like	Down-regulated

Superoxide dismutase (SOD) can catalyze the conversion of superoxide radicals to hydrogen peroxide and molecular oxygen, playing a key role in the protection of cell injury induced by oxygen free radicals. The expression of *SOD* (GRMZM2G169890 and GRMZM2G058522) in maize immature embryos cultured on the medium with cysteine was up-regulated (Table 3), compared with those without cysteine treatment. The addition of cysteine to the maize cultures after *Agrobacterium* infection could help to maintain the cell health in immature embryos, thus increasing transformation efficiency.

Glycosyltransferase is a transfer enzyme involved in glycosylation, playing important roles in the environmental adaptation of plants. The upregulation of several glycosyltransferase genes (GRMZM2G120016, GRMZM2G052571, and GRMZM5G834303) was seen in maize embryos (Table 3). By up-regulating the expression of these transfer enzymes, the immature embryo cells may be more vigorous after *Agrobacterium* infection, thereby improving the infection efficiency.

#### Genes involved in membrane integrity and transport

Many shared down-regulated genes involved in transmembrane integrity and transport were found in the RNA-seq analysis. These genes were primarily members of the aquaporin family (GRMZM2G041980, GRMZM2G392975, GRMZM2G126582, GRMZM2G081843, GRMZM2G027098, GRMZM2G047368, GRMZM2G178693, GRMZM2G154628, and GRMZM2G060922; Table 3). Aquaporins are small membrane proteins that consist of six membrane-spanning α-helices connected by five loops (A to E) where the N and C termini face the cytosol (Murata et al., 2000). The down-regulation of these genes may change the permeability of the cell membrane.

#### Genes involved in the cell wall metabolism

The cell wall is the first line of protection defending against the invasion of pathogenic bacteria. Several genes related to the cell wall were differentially expressed in the maize embryos grown on medium containing cysteine. A total of 19 up-regulated DEGs and 40 down-regulated DEGs shared between HiII and Z31 maize lines were identified and mapped to be putatively involved in cell wall metabolism. Among the 19 up-regulated genes, four genes (GRMZM2G166944, GRMZM2G392125, GRMZM2G070271, and GRMZM2G026980) were xyloglucan endotransglycosylase/hydrolase (XTH)-related genes, one gene (GRMZM2G070322) was a hormone receptor protein gene, while the remainder were glycosyltransferases or isomerase. Among the 40 down-regulated genes, 10 genes (GRMZM2G021621, GRMZM2G094990, GRMZM2G414779, GRMZM2G339122, GRMZM2G368886, GRMZM2G148485, GRMZM2G082520, GRMZM2G013002, GRMZM2G342246, GRMZM2G021427) were expansin-related genes and five genes (GRMZM2G025231, GRMZM2G178025, GRMZM2G453565, GRMZM2G147687, GRMZM2G141911) were cellulose synthase-related genes (Table 3).

## Discussion

Establishing a high efficient maize transformation system is necessary for the investigation of gene function and development of commercial transgenic maize events. Antioxidants such as dithiothreitol (DTT) or cysteine added into co-cultivation medium has been shown to significantly improve maize transformation efficiency (Frame et al., 2002; Vega et al., 2008). In this study, we confirmed that cysteine could improve the *Agrobacterium* infection efficiency of inbred line Z31 and hybrid line HiII. We also showed that HiII maize embryos were more sensitive to *Agrobacterium* infection compared to Z31 embryos (Figure 1). Many reports have demonstrated that HiII can achieve high transformation efficiency (Frame et al., 2002; Vega et al., 2008). Inbred line Z31 is an elite inbred line in China, and its transformation efficiency is relatively lower than HiII (data not shown).

It was proposed that cysteine may act as an antioxidant to minimize cell death caused by *Agrobacterium* infection (Frame et al., 2002; Vega et al., 2008). To our surprise, the addition of cysteine in the co-cultivation medium significantly increased the accumulation of H_2_O_2_ and ${\text{O}}_{2}^{-}$ (Figure 3), indicating that cysteine may improve the *Agrobacterium* infection efficiency not just as an antioxidant, but with other mechanism. It has also been shown that high cysteine concentration decreased the proportion of embryos that can give rise to embryogenic callus (Frame et al., 2002; Vega et al., 2008). To investigate the mechanism underlying cysteine-influenced improved *Agrobacterium* infection efficiency, we performed transcriptome analysis on maize embryos. The sequencing depth for HiII was lower than that for Z31, whereas more reads of HiII were mapped to the B73 reference genome compared to Z31 (Table 1). The HiII is F_2_ embryos derived from an F_1_ plant originating from a cross between HiII parent A and HiII parent B, both progenitors resulting from a cross between inbred lines A188 and B73 (Armstrong et al., 1991). Therefore, it was reasonable that more reads of HiII were mapped to the B73 reference genome than from Z31. Transcriptome profiling analysis showed that the cysteine treatment led to more up-regulated and down-regulated genes in HiII embryos than DEGs in Z31 embryos, confirming that HiII was more sensitive to the cysteine treatment than Z31. Numerous shared up-regulated and down-regulated genes were observed in HiII and Z31 embryos cultured on medium with cysteine, indicating that the cysteine treatment had a huge effect on the embryo cell metabolism, thus improving the efficiency of *Agrobacterium* infection. We also performed transcriptome analysis on the maize embryos treated with heat and found that few genes had changed expression levels compared to the untreated samples (data not shown), indicating that heat treatment might only induce cell competent prior to transformation.

The interaction between *Agrobacterium* and plant cells during *Agrobacterium* infection induces major changes in plant expression. *Agrobacterium* infection upregulated the expression of some plant genes, but also inhibited the expression of some host defense genes. In Arabidopsis tissues infected by *Agrobacterium*, the DEGs are mainly involved in photosynthesis, carbohydrate metabolism, cell wall synthesis, carbon and nitrogen metabolism, etc. (Deeken et al., 2006). In this study, our purpose was to investigate the mechanism behind cysteine-exposed infection efficiency improvement in maize embryos. The cysteine treatment up-regulated 939 genes and down-regulated 549 genes in both Z31 and HiII, respectively (Figure 4). The up-regulated genes were mainly involved in the oxidation reduction process, whereas the down-regulated genes were mainly involved in cell wall metabolism.

Plants will have an oxidative burst with high ROS accumulation upon pathogen attack, resulting in programmed cell death (PCD) and cellular defense response (Heller and Tudzynski, 2011; O'brien et al., 2012). To detoxify the oxidative stress, plant cells have several enzymatic and non-enzymatic systems. Among the common up-regulated genes in HiII and Z31 maize embryos, many were involved in the oxidation-reduction process or oxidoreductase activity process and metabolic process (Table 3). Cysteine synthase (GRMZM2G036708), an enzyme responsible for the final step in cysteine biosynthesis, was also upregulated in maize embryos after *Agrobacterium* infection, indicating that plant cells might need more antioxidants, i.e., cysteine, to cope with the oxidative stress. SOD can catalyze the conversion of superoxide radicals to hydrogen peroxide and molecular oxygen, playing a key role in the protection of cell injury induced by oxygen free radicals. The expression of *SOD* (GRMZM2G169890 and GRMZM2G058522) in maize immature embryos cultured on the medium with cysteine was up-regulated (Table 3) compared to those without cysteine treatment. This may be helpful to maintain the cellular homeostasis of immature embryos, increasing the efficiency of *Agrobacterium* infection. The up-regulation of several glycosyltransferase genes (GRMZM2G120016, GRMZM2G052571, and GRMZM5G834303) in maize embryos was also observed (Table 3), that might participate in the regulation of redox status. Glycosyltransferase is a kind of transfer enzyme involved in glycosylation. It has been shown that several Arabidopsis glycosyltrasferase can regulate the redox status and detoxify the ROS (Simon et al., 2014).

We found that the addition of cysteine in the co-cultivation medium significantly decreased cell wall-related protein occurrence. This indicated that the addition of cysteine improved the *Agrobacterium* infection efficiency not only by increasing expression of detoxifying enzymes but also affecting the expression of genes involved in the cell membrane and cell wall metabolism. Aquaporin proteins are multifunctional and act as a selective channel protein for water passage as well as in the physiological processes of other material transport, cell elongation and differentiation (Chaumont and Tyerman, 2014). Several aquaporin genes were down-regulated in the maize embryos treated with cysteine. The down-regulation of these genes may change the permeability of the cell membrane and increase water channel activity, providing favorable conditions for the invasion of *Agrobacterium*.

The cell wall is the initial grounds of defense against pathogenic bacteria invasion. Several genes related to the cell wall were up-regulated or down-regulated in maize embryos grown on medium containing cysteine. Xyloglucan endotransglucosylase/hydrolase (XTH) is a cell wall loosening enzyme and plays a key role in relaxing the cell wall (Rose et al., 2002; Van Sandt et al., 2007). The expression of *XTH* is significantly increased during the ripening of Kiwifruit (Redgwell and Fry, 1994) and tomato (Miedes and Lorences, 2009), indicating that XTH is involved in cell wall degradation, resulting in ripened fruit that turn soft upon maturity. When overexpressing *ZmXTH1* in Arabidopsis, the activity of glucan hydrolase increases and the structure and composition of the cell wall significantly change in transgenic Arabidopsis (Genovesi et al., 2008). In this study, the up-regulated expression of cell wall integrity-related genes such as, XTH, glucose isomerase, and isomerase may have affected the maize embryo cell wall structure, thus altering the infection efficiency.

Expansins are plant cell wall-loosening proteins and encoded by multigene families in land plants (Javier and Cosgrove, 2005). It was found that the down-regulated expression of expansin gene inhibits the growth and development of plants (Pien et al., 2001). In most cases, the overexpression of expansion genes can stimulate the plant cell growth (Ma et al., 2013). It is proposed that expansion proteins can anchor the surface of cellulose, thus being able to detach glucans from the cellulose surface, whereas it is also possible that expansins can decrease the activity of some cell wall degrading enzymes (Nardi et al., 2013). In this study, the down-regulation of several expansion genes may stimulate the cell wall degradation. Cellulose synthase is a key enzyme in cell wall synthesis and 12 genes have been identified in maize (Appenzeller et al., 2004). It has been shown that mutation of a cellulose synthase-like gene inhibits the *Agrobacterium* transformation of Arabidopsis root (Zhu et al., 2003a). Together, the change in cellulose synthase and expansin genes induced by the cysteine treatment may lead to the high cell wall elasticity and slow cell proliferation. This would keep the cell wall in a relatively permeable state so as to improve the infection efficiency of the embryos.

## Conclusion

The addition of cysteine in the co-cultivation medium significantly increased the *Agrobacterium* infection efficiency on maize embryos. The addition of cysteine in the co-cultivation medium significantly increased the accumulation of ROS in embryos, indicating that cysteine may improve the *Agrobacterium* infection efficiency not just as an antioxidant. Transcriptome profiling analysis revealed that the addition of cysteine induced up-regulation in genes mainly involved in the oxidation reduction process, whereas the down-regulated genes were mainly involved in cell wall metabolism. We hypothesize that cysteine could loosen the cell wall by modifying cell wall and membrane metabolism during *Agrobacterium* infection, thereby improving the entry of *Agrobacterium* into plant cells.

## Author contributions

YL, JW, and YjL designed the research. YL performed the research. ZZ, YL, JF, GW, and YjL analyzed the data. YL, JW, and YjL wrote the article.

### Conflict of interest statement

The authors declare that the research was conducted in the absence of any commercial or financial relationships that could be construed as a potential conflict of interest.

## Availability of supporting data

The sequencing data were deposited in the NCBI Sequencing Read Archive (SRA) with accession number SRR5980095, SRR5980096, SRR5979992, SRR5980097, SRR5980098, SRR5980099, SRR5980100, SRR5980101, SRR5980102, SRR5980105, SRR5980110, SRR5980111, SRR5980112, SRR5980113, SRR5980132, and SRR5980133.
