# Corrigendum: Transcriptome Analysis of Maize Immature Embryos Reveals the Roles of Cysteine in Improving *Agrobacterium* Infection Efficiency

**DOI:** 10.3389/fpls.2017.01964

**Published:** 2017-11-07

**Authors:** Yan Liu, Zhiqiang Zhang, Junjie Fu, Guoying Wang, Jianhua Wang, Yunjun Liu

**Affiliations:** ^1^College of Agronomy and Biotechnology, China Agricultural University, Beijing, China; ^2^Institute of Crop Sciences, Chinese Academy of Agricultural Sciences, Beijing, China

**Keywords:** *Agrobacterium*, infection efficiency, cysteine, maize embryo, transcriptome

In the original article, there was a mistake in Table 3 as published. In original Table 3, four aquaporin genes (GRMZM2G041980, GRMZM2G392975, GRMZM2G126582, GRMZM2G081843) were repeated two times, and we neglected to include several upregulated glycosyltransferase genes (GRMZM2G120016, GRMZM5G834303). The corrected Table 3 appears below.

**Table 3:** DEGs which involved in the cell wall metabolism.

**Pathway**	**Gene ID**	**Gene anotation**	**Expression pattern**
	GRMZM2G120016	Cis-zeatin O-glucosyltransferase	Up-regulated
	GRMZM5G834303	Cytokinin-O-glucosyltransferase 2	Up-regulated
Oxidation reduction process	GRMZM2G052571	Glutathione S-transferase	Up-regulated
	GRMZM2G056388	Glutathione S-transferase	Up-regulated
	GRMZM2G036708	Cysteine synthase	Up-regulated
	GRMZM2G159587	Glyoxylate reductase	Up-regulated
	GRMZM2G170017	Carbonyl reductase 1	Up-regulated
	GRMZM2G177077	Glucose-6-phosphate 1-dehydrogenase	Up-regulated
	GRMZM2G141473	Aldehyde oxidase-2	Up-regulated
	GRMZM2G169890	Superoxide dismutase	Up-regulated
	GRMZM2G058522	Superoxide dismutase	Up-regulated
	GRMZM2G471357	Peroxidase 52	Up-regulated
	GRMZM2G440208	6-phosphogluconate dehydrogenase	Up-regulated
	GRMZM2G173195	Glycerol-3-phosphate dehydrogenase	Up-regulated
	GRMZM2G090980	Mannitol dehydrogenase	Up-regulated
	GRMZM2G058244	UDP-glucose 6-dehydrogenase	Up-regulated
	GRMZM2G053720	Proline oxidase	Up-regulated
	GRMZM2G074743	Alternative oxidase	Up-regulated
	GRMZM2G479423	Aldose reductase	Up-regulated
	GRMZM2G443445	Mannitol dehydrogenase	Up-regulated
	GRMZM2G099467	Gibberellin 20 oxidase 2	Up-regulated
	GRMZM2G072529	Acc oxidase	Up-regulated
	GRMZM2G102959	Ferredoxin–nitrite reductase	Up-regulated
Membrane Integrity and transport	GRMZM2G041980	Aquaporin NIP1-1	Down-regulated
	GRMZM2G392975	Aquaporin PIP1-1	Down-regulated
	GRMZM2G126582	Aquaporin NIP-type	Down-regulated
	GRMZM2G081843	Aquaporin PIP 1-3	Down-regulated
	GRMZM2G027098	Aquaporin TIP2-2	Down-regulated
	GRMZM2G047368	Aquaporin PIP2-4	Down-regulated
	GRMZM2G178693	Aquaporin PIP2-4	Down-regulated
	GRMZM2G154628	Aquaporin PIP2-4	Down-regulated
	GRMZM2G060922	Aquaporin SIP1-2	Down-regulated
	GRMZM2G159632	Sulfate transporter	Down-regulated
	GRMZM2G442523	Sugar transport protein 5	Down-regulated
	GRMZM2G063824	Carbohydrate transporter	Down-regulated
	GRMZM2G342907	Sulfate transporter	Down-regulated
	GRMZM2G036448	Amino acid-polyamine transporter	Down-regulated
Cell wall metabolism	GRMZM2G166944	xyloglucan endotransglucosylase	Up-regulated
	GRMZM2G392125	xyloglucan endotransglucosylase	Up-regulated
	GRMZM2G070271	probable xyloglucan endotransglucosylase	Up-regulated
	GRMZM2G026980	xyloglucan endotransglycosylase	Up-regulated
	GRMZM2G070322	systemin receptor SR160	Up-regulated
	GRMZM2G021621	expansin-B4	Down-regulated
	GRMZM2G094990	beta-expansin 1a	Down-regulated
	GRMZM2G414779	expansin-A31-like	Down-regulated
	GRMZM2G339122	alpha expansin 1	Down-regulated
	GRMZM2G368886	alpha expansin 4	Down-regulated
	GRMZM2G148485	expansin-B15-like	Down-regulated
	GRMZM2G082520	beta-expansin 1a	Down-regulated
	GRMZM2G013002	beta expansin 8	Down-regulated
	GRMZM2G342246	beta-expansin 7	Down-regulated
	GRMZM2G021427	expansin-B3-like	Down-regulated
	GRMZM2G025231	cellulose synthase 7	Down-regulated
	GRMZM2G178025	endoglucanase 12-like	Down-regulated
	GRMZM2G453565	endoglucanase 2-like	Down-regulated
	GRMZM2G147687	exoglucanase 1	Down-regulated
	GRMZM2G141911	endoglucanase 4-like	Down-regulated
	GRMZM2G131912	pectate lyase 8	Down-regulated
	GRMZM2G091191	brassinosteroid-regulated protein BRU1-like	Down-regulated

In the original article, there was an error. The reads from the HiII embryo samples were incorrectly presented as “7,432,161–15,904,122”. The correct reads from the HiII embryo samples should be “7,432,161–15,881,554” as shown in **Table 1**.

A correction has been made to (Results), (Maize Embryo Transcriptome Profiling), (Paragraph 1):

To investigate the mechanism of cysteine-related transformation efficiency improvement, we performed transcriptome analysis of the maize embryos cultured on the medium with or without cysteine. The experiment was performed with four independent biological replicates. We obtained 7,432,161–15,881,554 reads from the HiII embryo samples and more than 78.73% of the reads were mapped to the B73 reference genome. We obtained 20,119,176–24,789,278 reads from the inbred line Z31 embryo samples and 73.66% of the reads were mapped to the B73 reference genome (**Table 1**). Maize has ~ 30,000 genes and the sequencing depth we acquired was enough for subsequent analysis. The authors apologize for this error and state that this does not change the scientific conclusions of the article in any way.

## Conflict of interest statement

The authors declare that the research was conducted in the absence of any commercial or financial relationships that could be construed as a potential conflict of interest.

