# Supplementary material for: Transcriptome Analysis of Maize Immature Embryos Reveals the Roles of Cysteine in Improving Agrobacterium Infection Efficiency
Source: Front Plant Sci. 2017 Oct 17;8:1778. doi: 10.3389/fpls.2017.01778 (PMC5651077; doi:10.3389/fpls.2017.01778)
Supplement: Supplementary file 1 [file Table1.DOCX]

**Table S1 The primers used in qRT-PCR**

| **Gene** |  | **Primers** |
| --- | --- | --- |
| GRMZM2G036708 | sense | 5’-CCAGGAAAGACCACGTTGAT-3’ |
|  | anti-sense | 5’-TCTCCAGGCTGGTGTAGGAC-3’ |
| GRMZM2G170017 | sense | 5’-GCCGTCAAAGAAATGATGGT-3’ |
|  | anti-sense | 5’-GTGCCGTGGTAGTTGATCCT-3’ |
| GRMZM2G087875 | sense | 5’-AGGAACACCTACTCCGACGA-3’ |
|  | anti-sense | 5’-GGCACGTAGTTCCACAGGTT-3’ |
| GRMZM2G132875 | sense | 5’-TGCCTTTGTCTTCCCGTATT-3’ |
|  | anti-sense | 5’-CAGCCTCTCCCTGTTGTAGC-3’ |
| GRMZM2G074743 | sense | 5’-CTCAGGGCGTCTTCTTCAAC-3’ |
|  | anti-sense | 5’-GGTCCTTGAGGTACTCGGTGT-3’ |
| GRMZM2G443445 | sense | 5’-CTGGGGTTGTGACTGAGGTT-3’ |
|  | anti-sense | 5’-GGCACGAGTTCACGTAGGTT-3’ |
| GRMZM2G097641 | sense | 5’-CGATAGCTGGGAGATGGAAG-3’ |
|  | anti-sense | 5’-CCAGGTCTTGTGGATGTGTG-3’ |
| GRMZM2G070322 | sense | 5’-AGGAGTGCATCTCGTTCCTG-3’ |
|  | anti-sense | 5’-GAGCATGCATAGGTGGAGGT-3’ |
| GRMZM2G025105 | sense | 5’-CTCAACCAGCTGGACCTCTC-3’ |
|  | anti-sense | 5’-CCTTATCGAGCCTGCTGAAG-3’ |
| GRMZM2G166944 | sense | 5’-CATCCTCTGGAACCCCAAG-3’ |
|  | anti-sense | 5’-TCCACAGGCTGGAGTAGAGC-3’ |
| GRMZM2G025190 | sense | 5’-GCTACGAGAACGTGGAGGAG-3’ |
|  | anti-sense | 5’-CGTCGATGTACTGCAGGATG-3’ |
